# Free manipulation system for nanorobot cluster based on complicated multi-coil electromagnetic actuator

**DOI:** 10.1038/s41598-021-98957-y

**Published:** 2021-10-05

**Authors:** Yun Kim, Jun Keun Chae, Jong-Hwan Lee, Eunpyo Choi, Yoon Koo Lee, Jihwan Song

**Affiliations:** 1 Department of Mechanical Engineering, Hanbat National University, Daejeon, 34158 Republic of Korea; 2 Center for Convergent Research of Emerging Virus Infection, Korea Research Institute of Chemical Technology, Daejeon, 34114 Republic of Korea; 3 School of Mechanical Engineering, Chonnam National University, Gwangju, 61186 Republic of Korea

**Keywords:** Biomedical engineering, Computational science

## Abstract

Chemotherapy is an important method in the field of cancer treatment and often follows surgery and/or radiotherapy to remove as many tumor cells as possible. In particular, among the chemotherapy methods, treatment using electromagnetic-based actuation systems is considered an effective method owing to the remote control of nanorobots. The existing electromagnetic-based actuation systems, however, have certain disadvantages such as the lack of degrees of freedom and the difficulty of manipulating large numbers of nanorobots (i.e., nanorobot clusters). Herein, we report that nanorobot clusters can be manipulated with high degrees of freedom through a simple parameter alpha that easily controls the gradient of the magnetic field of a multi-coil electromagnetic actuation system. The simulation results show that the gradient of the magnetic field is controlled using an introduced parameter, alpha, and the corresponding velocity is also controlled. Not only the velocity of the nanorobot cluster but also the unrestricted spatial control is enabled in two- and three-dimensions. We believe this study highlights an efficient method of electromagnetic control for cluster-based drug delivery.

## Introduction

Cancer is one of the crucial diseases in the world. Various treatments, such as surgery, radiation therapy, and chemotherapy, have been studied to overcome it. Surgery is a general treatment because it can remove a large volume of the tumor. Radiation therapy can also kill a large proportion of tumor cells without pain or anesthesia. Chemotherapy follows surgery or radiation therapy to remove as many remaining tumor cells as possible. Chemotherapy has the advantage of killing tumor cells throughout the body and preserving skin or organs because a resection is not required. In addition, chemotherapy does not expose patients to high levels of radiation. However, conventional chemotherapy has certain disadvantages; for example, chemotherapy drugs kill normal and healthy cells unevenly^1–4^. To decrease the damage to healthy cells and concentrate on the tumor cells, a drug delivery system is proposed that can release the drugs selectively to the targeted tumor cells.

To deliver the drugs to the target, many actuation methods, including chemical propulsion, magnetic propulsion, acoustic propulsion, and biological propulsion^5–10^, have been investigated and developed to enhance the targeting efficacy. Among these methods, magnetic drug delivery systems have the advantage of remote controllability owing to the penetrability of the magnetic field^11–13^. As the magnetic field penetrates the human body, nanorobots (i.e., nanoscale magnetic particles) containing drugs can be delivered to the target by changing the magnetic field remotely. Due to the advantages of the magnetic propulsion method, chemotherapy with magnetic propulsion using a magnetic actuation system can be a practical method for cancer treatment.

As an early magnetic actuation system for a drug delivery system, permanent magnets have been used to generate a magnetic field owing to its simplicity and ease of use^14,15^. However, permanent magnetic actuation systems are unable to control the magnetic field easily because they must be physically moved to change the magnetic field. Moreover, they cannot be turned off during emergencies^16–18^. For these reasons, electromagnetic actuation systems have been proposed instead of permanent magnets. In comparison to permanent magnetic actuation systems, electromagnetic actuation systems can vary the magnetic field rapidly by adjusting the current. Furthermore, electromagnetic actuation systems can be controlled by blocking the current during emergencies.

To date, many electromagnetic actuation systems have been investigated due to their advantages^19–24^. Although researchers have proposed various electromagnet-based actuation systems, such systems still have some difficulty controlling the magnet particles. For instance, they only provided a restricted degrees of freedom due to the two-dimensional arrangement of electromagnets^19^ and fluid flow-assisted propulsion^20,21^. To ensure a high degrees of freedom, an electromagnetic actuation system that includes eight electromagnets is suggested^22^. In addition, to precisely control the magnetic force on the particles, they numerically calculated the current combination of eight coils at a particular position where the magnetic particles were placed. However, this approach requires the repeated update of the current combination using a pseudo-inverse matrix at every position where the magnetic particle exists. In particular, if many nanorobots are injected into the vessel at the same time (i.e., nanorobot cluster), the position of the nanorobots cannot be specified. From the viewpoint of drug delivery, cluster-based delivery has advantages over a single robot. Nanorobot clusters can swim into small spaces such as capillaries since they can deform their shapes with a variety^25^ while a single robot cannot swim into small spaces owing to their physical constraints (i.e., size). Besides, nanorobot clusters can load a greater amount of drugs than a single robot^26^ since they load the drug at their surface^27^ and have a larger surface area than a single robot of the same size. These advantages brought out various cluster-based manipulation studies^25,26,28,29^. However, these systems include the complicated process to manipulate the nanorobot clusters, which can decrease the delivery efficiency. Furthermore, in previous studies, the magnetic field gradient is overlooked, despite being one of the important variables that affect the magnetic force. The uniform magnetic force exerted to the nanorobot clusters would be very important for cluster-based manipulation because the nanorobot clusters are spatially distributed, not a single point. If the magnetic gradient is not uniform, the nanorobot clusters would result in undesirable situations such as separation of clusters and non-simultaneous movement. Besides, the locomotion of nanorobot clusters can be expected without difficulty since nanorobot clusters would travel under the uniform magnetic force.

In this study, we present the manipulation of nanorobot clusters with a high degrees of freedom under a complex multi-coil electromagnetic actuation system. To ensure a free manipulation, a simple parameter for the eight-coil electromagnetic actuator is introduced, which provides easy control of the magnetic field and its gradient. The simulation results show that a relatively uniform magnetic field gradient can be generated by exploiting the proposed parameter, irrespective of the intensity of the magnetic field in the channel. The average velocity of the nanorobot cluster was evaluated based on the results of the magnetic field study. The nanorobot cluster can be controlled with various velocities according to the intensity of the magnetic field and the parameter applied. In addition, the nanorobot cluster shows unrestricted spatial control with a simply controlled magnetic field by varying parameter as well as the velocity.

## Results and discussion

**Figure 1:**
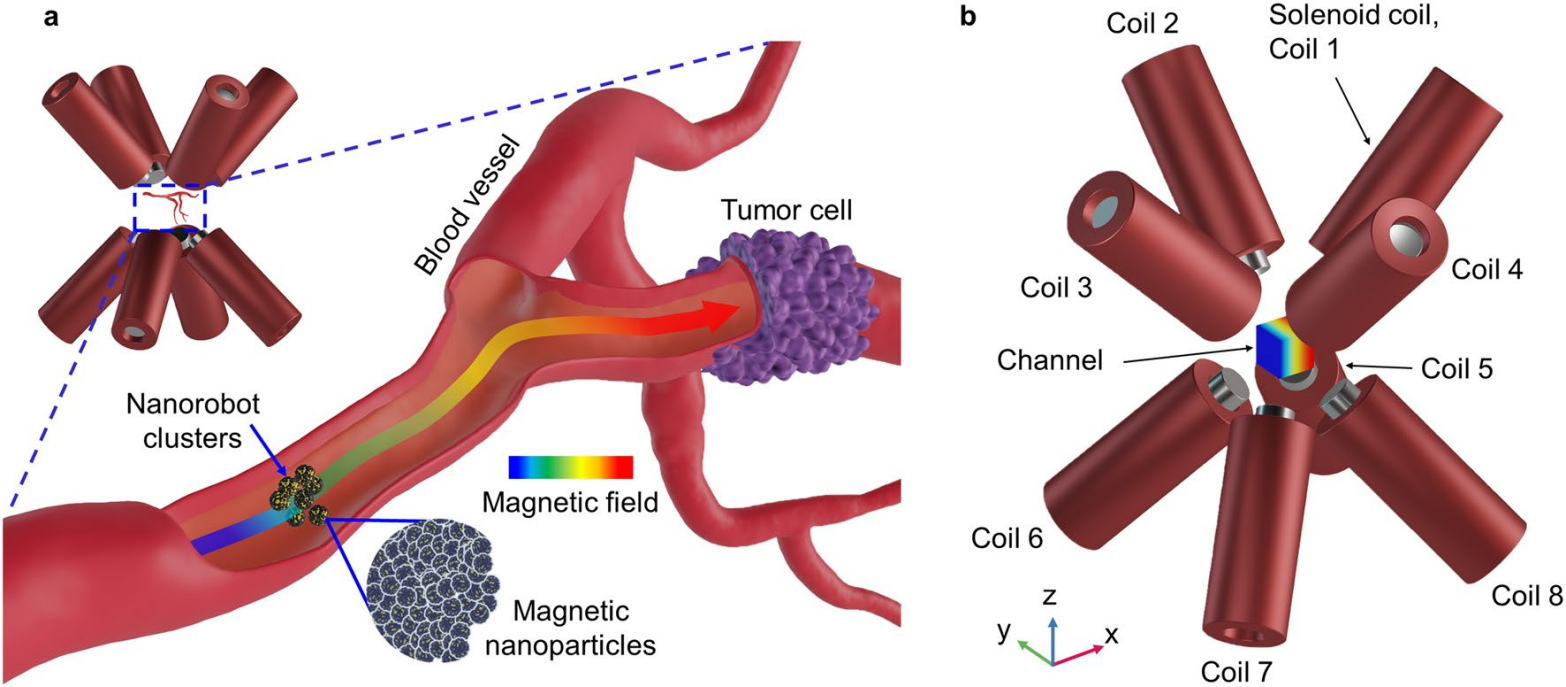
Schematic image of the magnetic drug delivery system and the electromagnetic actuation system. (**a**) Schematic illustration of the magnetic drug delivery system. The nanorobot cluster is pulled to the target due to the magnetic field gradient. (**b**) Schematic illustration of the electromagnetic actuation system with labelled coils. The electromagnetic actuation system includes coils, cores, and a channel.

To evaluate the behavior of the nanorobot cluster according to the induced magnetic field and its gradient, simulations on coupling the magnetic field calculation and particle tracing were conducted. All simulations were carried out using commercial software (COMSOL Multiphysics 5.4). Figure 1 shows a schematic illustration of the magnetic drug delivery system and the eight-coil electromagnetic actuation system considered here. A large number of nanorobots are injected into the blood vessel and controlled by the magnetic field gradient toward the target. The electromagnetic actuation system is composed of eight coils with cores inside to reinforce the intensity of the magnetic field. Cobalt and nickel steels are considered core materials. A cubic channel was placed at the center of the domain, where the coordination of the center was (0, 0, 0). The channel was filled with water, and the dimensions were 10 × 10 × 10 mm^3^. Properties of nanorobot clusters are based on our previous work^30^. Magnetization of nanorobot clusters and aggregated size were measured after synthesizing magnetite clusters, gold nanoparticles, Polydopamine (PDA), Polyethylene glycol (PEG), and Folic acid (FA).

### Control of magnetic field and gradient

The magnetic field, including the magnetic flux density and gradient, is studied through COMSOL Multiphysics 5.4, which is the driving force of motion of the nanorobot cluster. First, the current combinations for each coil used to form a magnetic field without a gradient are numerically calculated with pre-calculated magnetic flux density data for the unit current in the channel. To generate the gradient of the magnetic field, parameter alpha, α, is introduced. The range of parameter α was determined from the preliminary results of the magnetic field according to the current for the considered core material (Supplementary Fig. S1). The current combination is modified with parameter, α and it makes a uniform gradient of the magnetic field in the domain simply (details about the current combination are provided in “Methods”).

**Figure 2:**
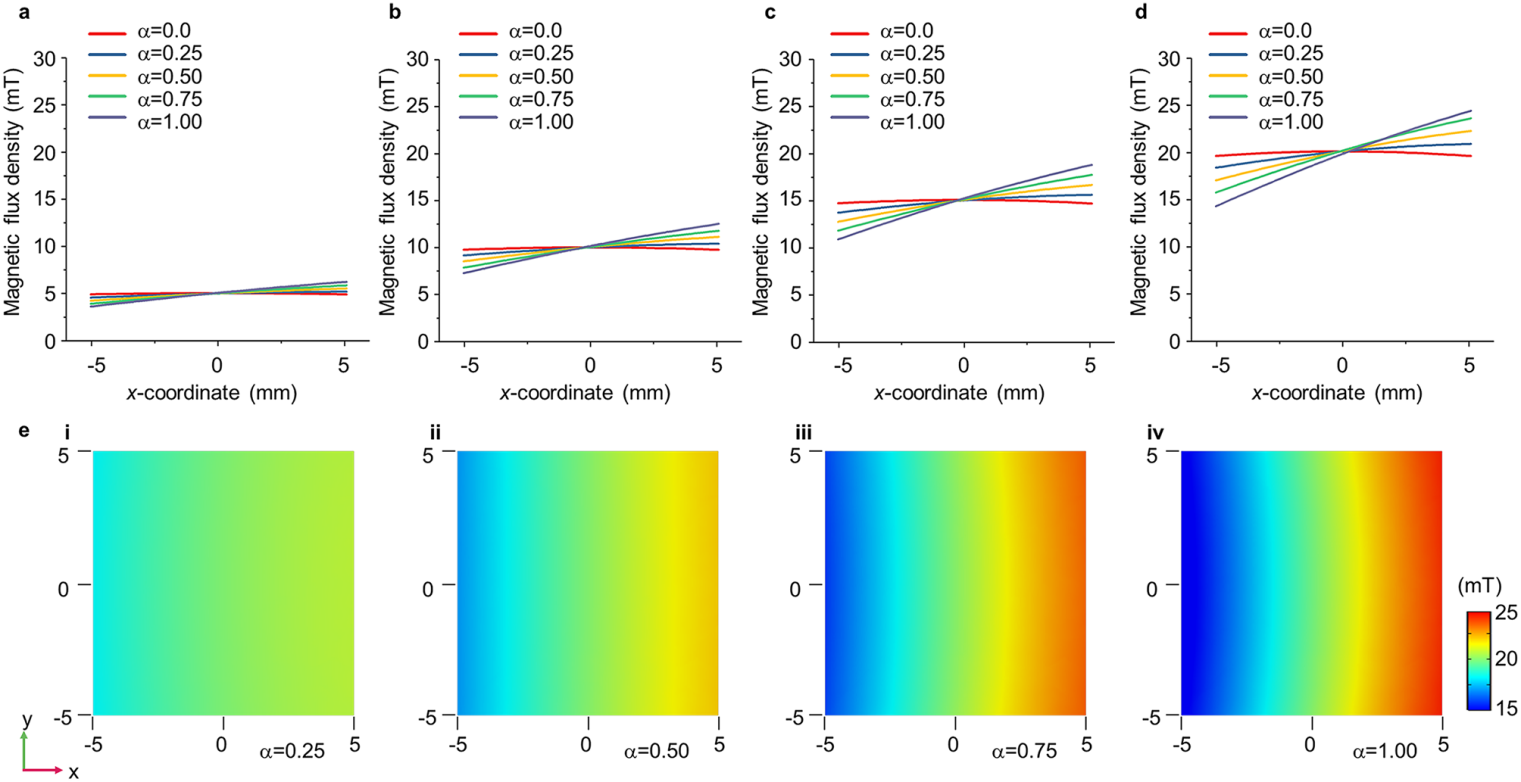
Controllable magnetic field in the channel. (**a**)–(**d**) The magnetic flux density profile in the channel along the *x-*axis with various values of α. The magnetic flux density at the center of the channel is set to (**a**) 5, (**b**) 10, (**c**) 15, and (**d**) 20 mT, respectively. (**e**) The magnetic field in the channel with a magnetic flux density of 20 mT for a parameter change from (i)–(iv) 0.25 to 1.00, respectively (COMSOL Multiphysics 5.4).

Figure 2 shows a controllable magnetic field with parameter α. Figure 2a–d shows the magnetic flux density along the *x-*axis in the channel. Four cases (i.e., magnetic flux densities of 5, 10, 15, and 20 mT at the center of the channel) were considered. For 5 mT, the magnetic flux density at the ends of the channel in the *x-*axis varies from 4.9 to 3.6 mT at the left end (i.e., at the position of − 5.0 mm) and to 6.2 mT at the right end (i.e., at the position of 5.0 mm) when parameter α changes from 0 to 1.00 (Fig. 2a). The gradient increased from 0 to 0.26 T/m. In the case of 10 mT, the magnetic flux density decreases from 9.8 to 7.3 mT at the left end of the channel, and increases to 12.5 mT at the right end of the channel with a change in α from 0 to 1.00 (Fig. 2b). For 10 mT, the gradient varies from 0 to 0.52 T/m. For 15 mT, the magnetic flux density decreases from 14.7 to 10.9 mT at the left end and increases to 18.8 mT at the right end (Fig. 2c). The gradient varies from 0 to 0.78 T/m when the magnetic flux density is 15 mT. Similarly, the magnetic flux density in the case of 20 mT shows a variation of 19.6–14.3 mT and 24.4 mT at each end of the channel, respectively (Fig. 2d). With a magnetic flux density of 20 mT, the gradient increases from 0 to 1.02 T/m. As α increases, the magnetic flux density decreases at the negative directional end and increases at the positive directional end, which implies an increase in the gradient. Figure 2e shows the magnetic field in the channel according to parameter α when the magnetic flux density at the center of the channel is 20 mT. As shown in Fig. 2e, the magnetic field is varied, and a relatively constant gradient is easily formed with the control of the parameter. A similar gradient of the magnetic field appeared in the *y-* and *z-*directions (Supplementary Fig. S2).

To show the adaptability for the formation of the magnetic field and its gradient with the parameter α, irrespective of the core materials, not only cobalt steel for core but nickel steels are also considered for the core. In the case of a nickel steel core, the magnetic flux density at the center of the channel is different (i.e., 2.5–10 mT) from that of the cobalt steel core because they have different magnetic properties. To generate the magnetic flux density and its gradient with the nickel steel core, α varies from 0 to 1.00 (Supplementary Fig. S3). Although cobalt steel and nickel steel have different magnetic properties, the gradient of the magnetic field can be simply formed using the proposed parameter. In the case of 2.5 mT, the gradient of the magnetic field obtained is 0, 0.04, 0.07, 0.10, and 0.13 T/m when α varies 0, 0.25, 0.50, 0.75, and 1.00, respectively. For 5, 7.5, and 10 mT, the gradient increases from 0 to 0.26, 0 to 0.39, and 0 to 0.52 T/m, respectively, with an increase in α from 0 to 1.00.

### Evaluation of velocity of nanorobot clusters

**Figure 3:**
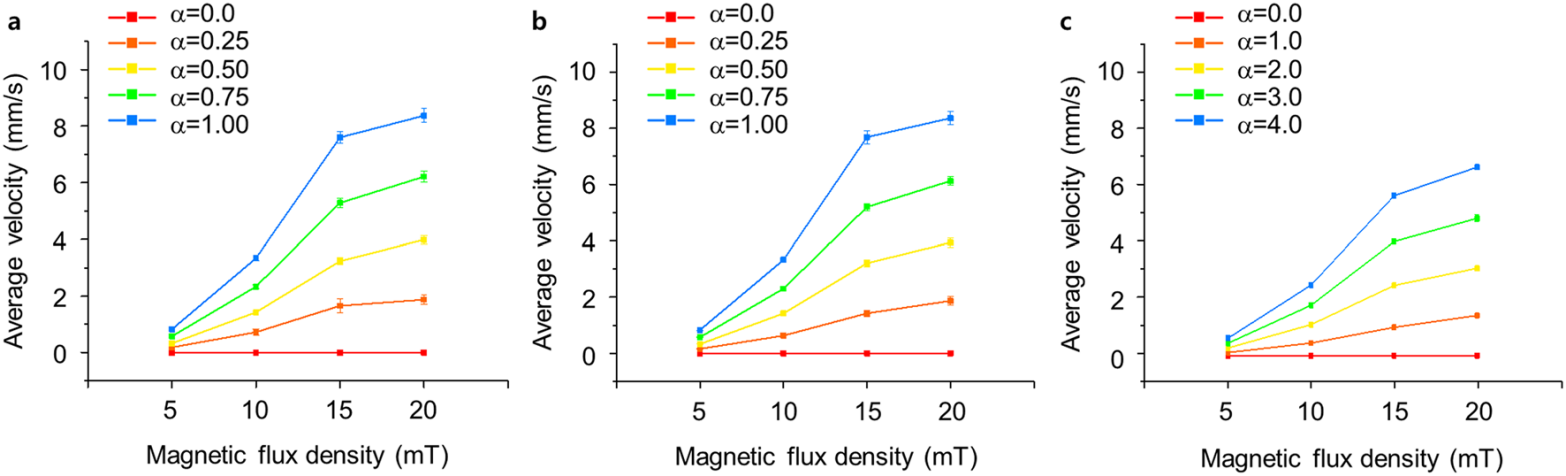
Average particle velocity according to the direction of motion. The average particle velocity in the (**a**) *x-*direction, (**b**) *y-*direction, and (**c**) *z-*direction where α is 0.00, 0.25, 0.50, 0.75, and 1.00 for the *x-* and *y-*directions and 0.0, 1.0, 2.0, 3.0, and 4.0 for the *z-*direction, and the magnetic flux density is 5, 10, 15, and 20 mT, respectively.

Based on the results of the magnetic field study, the average velocity of the nanorobot cluster under various conditions was evaluated using the particle tracing method using COMSOL Multiphysics 5.4. The nanorobot cluster (i.e., magnetic nanoparticles) was assumed to be a sphere 60 μm in diameter. The 500 clusters of spherical shape are released at the center of the channel within a radius of 0.5 mm. The average velocities of the nanorobot clusters in the *x-*, *y-*, and *z-*directions and their standard deviation with error bars are shown in Fig. 3a–c, respectively. The magnetic flux density varies from 5 to 20 mT, and the parameter α is also changed from 0.0 to 1.00 for the *x-* and *y-*directions, and from 0.0 to 4.0 for the *z-*direction to generate the gradient of the magnetic field. The average velocity of the nanorobot cluster in *x-*direction where the magnetic flux density is 5 mT is evaluated as 0, 0.2, 0.3, 0.6, and 0.8 mm/s with increase in α of 0.0 to 1.00 (Fig. 3a). The average velocity tends to increase with an increase in parameter α. In addition to a magnetic flux density of 5 mT, an increase in the average velocity according to the increase in parameter α is observed regardless of the intensity of the magnetic flux density. In the case of a magnetic flux density of 10 mT, the average nanorobot cluster velocity is 0, 0.7, 1.4, 2.3, and 3.3 mm/s where the α is 0, 0.25, 0.5, 0.75, and 1.00, respectively. The magnetic flux density of 15 mT shows a velocity of 0, 1.7, 3.2, 5.3, and 7.6 mm/s with an increase in α. In the case of 20 mT, with the same variations of α, the average velocity is obtained as 0, 1.9, 4.0, 6.2, and 8.4 mm/s.

For the *y-*direction, the average velocity is almost the same as that in the *x-*direction because the coils are aligned alike in the *x-*direction (Fig. 3b). For the *z-*direction, however, the coils are aligned unlike in the other directions. Even though the coil alignment in the *z-*direction is different from the *x-* and *y-*directions, parameter α can generate a gradient with an increase in α. Due to the different alignments, α varies from 0.0 to 4.0. The average velocity when the nanorobot cluster moves toward the *z-*axis is calculated as 0, 0.1, 0.3, 0.4, and 0.6 mm/s, where the magnetic flux density is 5 mT, and α is 0.0, 1.0, 2.0, 3.0, and 4.0 (Fig. 3c). In the case of the magnetic flux density of 10 mT, the average velocity is 0, 0.4, 1.1, 1.8, and 2.5 mm/s with an increase of α from 0.0 to 4.0. For 15 and 20 mT, the average velocity is obtained from 0 to 5.6 and 0 to 6.6 mm/s, respectively, where α is 0.0 and 4.0.

First of all, the velocities from the simulation are consistent with the experimentally observed velocity range of the nanorobot cluster^30^. As shown in the above results, the nanorobot cluster travels faster as the magnetic flux density increases. Furthermore, the nanorobot cluster also travels faster as α increases. In particular, regardless of the direction of motion and the magnitude of the magnetic flux density, the average velocity of the nanorobot cluster increases with an increase in α, because it means an increase in the gradient that influences the magnetic force.

### Unrestricted spatial control of nanorobot clusters

**Figure 4:**
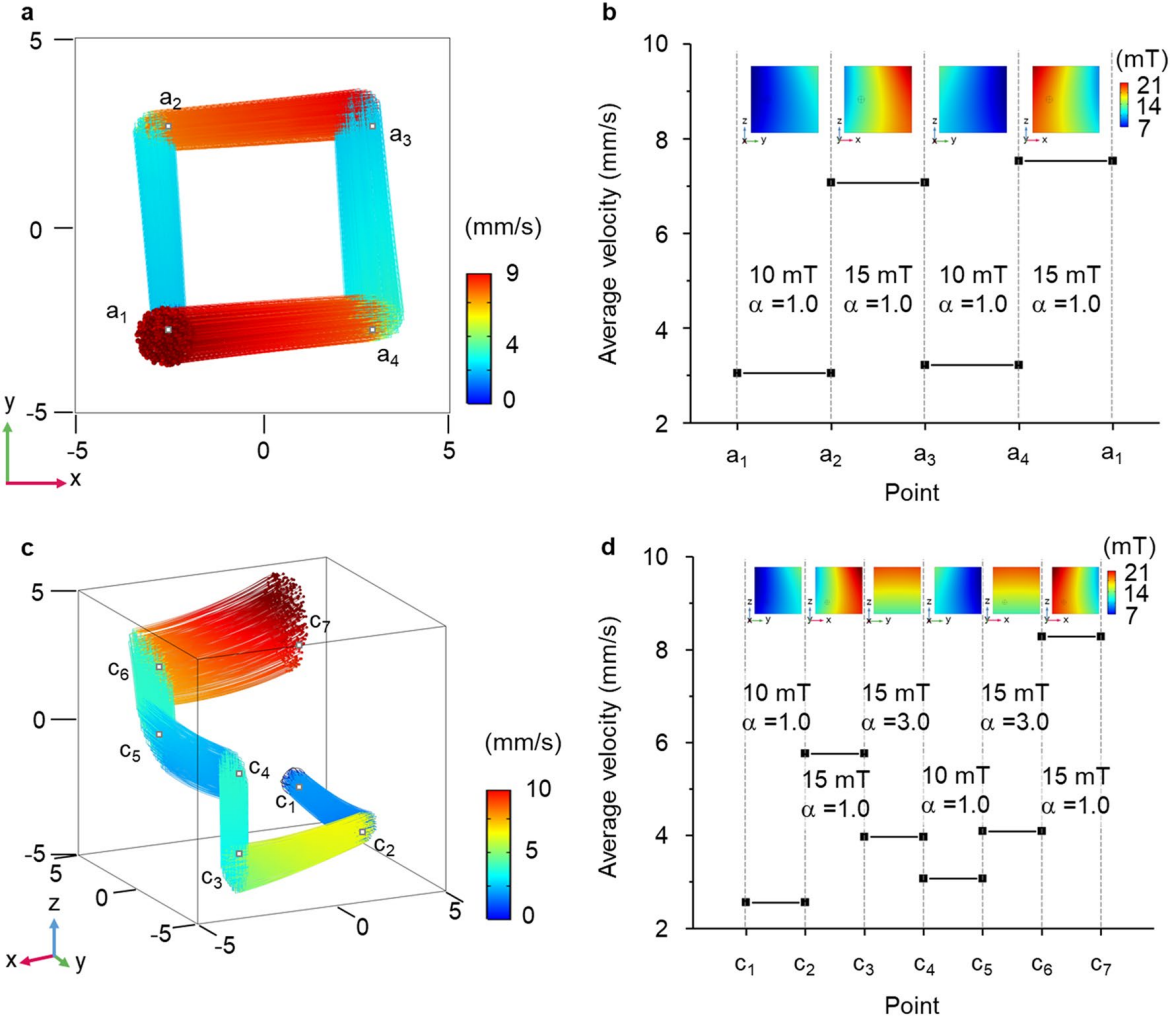
Spatial control of the nanorobot cluster with various magnetic fields according to the change in parameter α. (**a**) Two-dimensional control of nanorobot cluster. The colors represent the velocity of the nanorobot cluster. (**b**) The average velocity of the nanorobot cluster while traveling. (**c**) Three-dimensional control of nanorobot cluster. (**d**) The average velocity of the nanorobot cluster while traveling (COMSOL Multiphysics 5.4).

Figure 4 shows the unrestricted spatial control of the nanorobot clusters through COMSOL Multiphysics 5.4 with various magnetic field designs. Figure 4a shows the two-dimensional trajectories of the controlled nanorobot cluster from point a_1_ to a_4_ (Supplementary Video 1). The colors of the trajectories correspond to the velocities of the nanorobot cluster. The dimensions of the channel are 10 × 10 × 10 mm^3^. The average velocity of the nanorobot cluster during travel is shown in Fig. 4b. When 500 nanorobot clusters were initially released at a_1_ (− 2.5, − 2.5, 0), a magnetic field with an intensity of 10 mT and α = 1.0 was generated. The magnetic field is shown in the inset of Fig. 4b. As shown in Fig. 4a, the cluster moves along the *y-*axis with this magnetic field, and the nanorobot cluster travels with an average velocity of approximately 3.1 mm/s. To move the nanorobot cluster toward the next point (i.e., a_2_ to a_3_), the magnetic field was changed to an intensity of 15 mT and α = 1.0 (inset of Fig. 4b). The nanorobot cluster travels with an average velocity of approximately 7.1 mm/s. To reach the next point a_4_, a magnetic field of 10 mT and α = 1.0 (inset of Fig. 4b) is applied and the average velocity of the cluster is 3.2 mm/s in this section. Here, 15 mT and α = 1.0, are applied (inset of Fig. 4b) to move the cluster back to the starting point a_1_. The average velocity is approximately 7.5 mm/s while traveling.

In addition, three-dimensional control of the nanorobot cluster was performed from point c_1_ to c_7_ (Fig. 4c and Supplementary Video 2). The coordinates of each point, c_1_ to c_7_, are (− 2.5, − 2.5, − 2.5), (− 2.5, 2.5, − 2.5), (2.5, 2.5, − 2.5), (2.5, 2.5, 0), (2.5, − 2.5, 0), (2.5, − 2.5, 2.5), and (− 2.5, − 2.5, 2.5), respectively, and the dimensions of the channel are the same. Figure 4c shows the trajectories of the nanorobot clusters with colored lines representing their velocities. The average velocity of the nanorobot cluster according to this point is shown in Fig. 4d. Similarly, 500 nanorobot clusters were initially released at c_1_. The magnetic field continuously changes to manipulate the nanorobot cluster. First, an intensity of 10 mT and α = 1.0 is applied to move the nanorobot cluster from c_1_ to c_2_. The average velocity of the nanorobot cluster is approximately 2.6 mm/s during c_1_ to c_2_. The magnetic field was changed to an intensity of 15 mT and α = 1.0 to move the nanorobot cluster from c_2_ to c_3_. When the nanorobot cluster is heading to c_3_, their average velocity is approximately 5.8 mm/s. After passing point c_3_, the nanorobot cluster is pulled along the *z-*axis heading to c_4_, experiencing a magnetic field with an intensity of 15 mT and α = 3.0. The average velocity is approximately 4.0 mm/s. In the section of c_4_ to c_5_, the magnetic field changed to an intensity of 10 mT and α = 1.0. The nanorobot cluster travels along the *y-*axis with a velocity of 3.1 mm/s on average. To move the nanorobot cluster to the next point c_6_, the magnetic field was changed to 15 mT and α = 3.0. During travel, the average velocity is approximately 4.1 mm/s. Finally, heading to the last point c_7_, the magnetic field was changed to 15 mT and α = 1.0. In this section, the average velocity of the nanorobot cluster is approximately 8.3 mm/s. The magnetic fields in each section are plotted in the inset of Fig. 4d. Similarly, the unrestricted spatial control of nanorobot clusters is also performed with nickel core to analyze the behavior of nanorobot clusters under different magnetic fields (Supplementary Fig. S4). The nanorobot clusters under the magnetic field formed by nickel core also show unrestricted spatial movements but they show slower velocity compared to that of cobalt steel due to the early magnetic saturation of nickel core (Supplementary Fig. S1). In addition, nanorobot clusters can be spread for the release of drugs (Supplementary Fig. S5).

The results show that an unrestricted spatial manipulation and velocity can be achieved with simple parameter control, irrespective of the core materials. The magnetic fields are appropriately varied with the change in parameter α. As a result, the nanorobot cluster travels along the designated route.

## Conclusion

In this paper, a simple and easy way to manipulate nanorobot clusters was reported using a numerical simulation. To control nanorobot clusters, not a single nanorobot, control of the gradient of the magnetic field becomes significantly important as well as the intensity of the magnetic field. The current combination obtained using a pseudo-inverse matrix is modified by introducing parameter α to generate the gradient of the magnetic field. As a result, a relatively constant gradient was formed through the channel regardless of the direction, intensity of the magnetic fields, and core materials. Based on the results of magnetic field studies conducted according to parameter α, the average velocity of the nanorobot cluster was evaluated. The simulation results showed that the velocity of the nanorobot cluster can be controlled using the designed gradient of the magnetic field by parameter α. Furthermore, to demonstrate the possibility of unrestricted spatial control of the nanorobot cluster, as well as the velocity, two- and three-dimensional control of the nanorobot cluster was carried out. As shown in the simulation results, the free manipulation of the nanorobot cluster is accomplished in three-dimensions, not only in two-dimensions. The nanorobot clusters were fully controllable with the parameter we introduced, including the direction of motion and velocity, irrespective of the core materials. We believe that our method would provide a guide for an effective way to manipulate clusters. In addition, more practical manipulation and delivery would be accomplished if monitoring equipment such as computerized tomography (CT) and magnetic resonance imaging (MRI) are combined with our method in the experiments^30–32^.

## Methods

### Governing equations

The nanorobot cluster under an irregular magnetic field experiences a magnetic force, which is given by

1 $${\overrightarrow{{\varvec{F}}}}_{{\varvec{m}}}=\left(\overrightarrow{{\varvec{m}}}\bullet \nabla \right)\overrightarrow{{\varvec{B}}}=V\chi /{\mu }_{0}(\overrightarrow{{\varvec{B}}}\bullet \nabla )\overrightarrow{{\varvec{B}}},$$

where ${\overrightarrow{{\varvec{F}}}}_{{\varvec{m}}}$, $\overrightarrow{{\varvec{m}}}$, $\overrightarrow{{\varvec{B}}}$**,** $V$, $\chi$, ${\mu }_{0}$, and $\nabla$ represents the magnetic force, magnetic moment of the nanorobot cluster, magnetic field, volume of nanorobot cluster, magnetic susceptibility, magnetic permittivity of free space, and gradient, respectively.

The drag force also acts on the nanorobot cluster because they have velocity when they are pulled by an irregular magnetic field. Because a nanorobot cluster is assumed to be a sphere, the drag force follows Stokes’ law as follows:

2 $${\overrightarrow{{\varvec{F}}}}_{d}=6\pi \eta r\overrightarrow{{\varvec{u}}},$$

where ${\overrightarrow{{\varvec{F}}}}_{{\varvec{d}}}$, $\eta$, $r$, and $\overrightarrow{{\varvec{u}}}$ represent the drag force, viscosity of the surrounding fluid, radius of the nanorobot cluster, and velocity of the nanorobot cluster, respectively.

Consequentially, the equation of motion of the nanorobot can be described as

3 $$\sum \overrightarrow{{\varvec{F}}}=m\overrightarrow{{\varvec{a}}}=m\left(\frac{d\overrightarrow{{\varvec{v}}}}{dt}\right)={\overrightarrow{{\varvec{F}}}}_{m}+{\overrightarrow{{\varvec{F}}}}_{d},$$

where $m$, $t$, $\overrightarrow{{\varvec{v}}}$, and $\overrightarrow{{\varvec{a}}}$ represent the mass of the nanorobot cluster, time, the velocity of the nanorobot cluster, and the acceleration of the nanorobot cluster.

### Specifications of domain

During the numerical simulation, the electromagnetic actuation system was constituted as follows^30^: The diameter and length of the core were 22 and 123 mm, respectively. The coil wrapped the core 828 times. The inner and outer diameters of the coil were 24 and 48 mm, respectively, and their lengths were 120 mm. The core was far from the center of the channel, by up to 45.5 mm. The channel is considered as a cube with dimensions of $10\times 10\times 10 {\mathrm{mm}}^{3}$ to manipulate the nanorobot cluster in many different directions. Coils 1 through 4 (i.e., upper coils) were oriented at 90° intervals around the *z-*axis and tilted 45° from the *xy-*plane. Coils 5 through 8 (i.e., lower coils) were symmetrical to coils 1 through 4 and rotated 45° around the *z-*axis. The nanorobot cluster is considered to be a sphere 60 μm in diameter.

The coil was made of copper. The core material was chosen as the cobalt steel (VACOFLUX 50), and nickel steel was also considered to show the irrelevance of the core material. The channel is full of water, and the atmosphere is considered to be air. Nanorobot clusters are considered composed of magnetite-based materials, which are studied in our previous work^30^. Magnetization and size of nanorobot clusters were measured after synthesizing magnetite clusters, gold nanoparticles, Polydopamine (PDA), Polyethylene glycol (PEG), and Folic acid (FA).

### Current combination

To form the desired magnetic field at a certain point, it is useful to follow the method described below using a pseudo-inverse matrix. The matrices used in this calculation depend on the number of coils. Eight coils were considered in this study, and the matrices were based on it. Before the calculation, the magnetic field data generated by each coil with a unit current (1 A) at the point is required. The magnetic field data are evaluated at the center of the channel [i.e., coordinate (0, 0, 0)] in this study. The final magnetic field is formed by a superposition of every magnetic field formed by each coil. Thus, the final magnetic field at point can be expressed as follows:

4 $$\overrightarrow{{\varvec{B}}}=\stackrel{\sim }{{\varvec{B}}}{\varvec{I}},$$

where a $8\times 1$ matrix ${\varvec{I}}$ represents the applied current to the coils and a $3\times 8$ matrix $\stackrel{\sim }{{\varvec{B}}}$ represents the magnetic field generated by the unit current at the point. Matrix $\overrightarrow{{\varvec{B}}}$ is the final magnetic field at the point. In addition, the magnetic field and force at point can be expressed as follows:

5 $$\left[\begin{array}{c}\overrightarrow{{\varvec{B}}}\\ \overrightarrow{{\varvec{F}}}\end{array}\right]=\left[\begin{array}{c}\stackrel{\sim }{{\varvec{B}}}\\ {\overrightarrow{{\varvec{m}}}}^{T}\frac{\partial \stackrel{\sim }{{\varvec{B}}}}{\partial x}\\ {\overrightarrow{{\varvec{m}}}}^{T}\frac{\partial \stackrel{\sim }{{\varvec{B}}}}{\partial y}\\ {\overrightarrow{{\varvec{m}}}}^{T}\frac{\partial \stackrel{\sim }{{\varvec{B}}}}{\partial z}\end{array}\right]{\varvec{I}}={\varvec{A}}{\varvec{I}},$$

where $3\times 8$ matrices $\partial \stackrel{\sim }{{\varvec{B}}}/\partial x$, $\partial \stackrel{\sim }{{\varvec{B}}}/\partial y$, and $\partial \stackrel{\sim }{{\varvec{B}}}/\partial z$ represent the magnetic field gradients in the *x-, y-,* and *z-*directions at the point with the unit current, respectively. In addition, $\overrightarrow{{\varvec{m}}}$ and $\overrightarrow{{\varvec{F}}}$ represent the magnetic moment of the nanorobot and the magnetic force acting on the nanorobot cluster. Matrix ${\varvec{I}}$ can be calculated by multiplying the inverse matrix of ${\varvec{A}}$ with the left-hand side of the equation*.* However, since matrix ${\varvec{A}}$ is not a regular matrix, a pseudo-inverse matrix is used. The current combination matrix ${\varvec{I}}$ is expressed as follows:

6 $${\varvec{I}}={{\varvec{A}}}^{+}\left[\begin{array}{c}\overrightarrow{{\varvec{B}}}\\ \overrightarrow{{\varvec{F}}}\end{array}\right],$$

where ${{\varvec{A}}}^{+}$ represents the pseudo-inverse matrix of ${\varvec{A}}$*.* To obtain a current combination that forms the desired magnetic field without a gradient, the desired value of matrix $\overrightarrow{{\varvec{B}}}$ is set, and the magnetic force is set to zero in this study to avoid forming the gradient.

### Gradient formation

The current combination obtained by following the aforementioned method has a zero-gradient as the magnetic force is set to zero. To induce the magnetic force, the gradient must be formed, and the parameter α is introduced to ease the gradient formation. The coils are grouped in the same direction to clarify the effect of parameter α, which controls the current for the gradient. For example, to form the gradient in the *x-*direction, coils 1, 3, 5, 6, 7, and 8 are placed in the *x-*direction (Fig. 1), and they are separated into two groups, coils 1, 5, and 8, and coils 3, 6, and 7, because these two groups are heading in different directions (i.e., the positive and negative directions of the *x-*axis). We refer to these groups as groups 1 and 2, respectively. The coils in group 1 and group 2 have the same magnitude of current with the opposite sign when the domain is set to zero-gradient. To form the gradient in the positive *x-*axis, the coils in group 1 increase their current intensity, and those in group 2 decrease. We defined the value of parameter α multiplied by the current applied on coil 1 as the reference for the current change, and is added to the current on coil 1 from the zero gradient current. As the current on coil 1 is added by as much as ${\alpha i}_{1}$, it is subtracted from the current on the other coils (i.e., coils 5 and 8) as much as half this amount. These currents on coils 5 and 8 eliminate the gradient in the *z*-axis generated by the increase in current on coil 1. For the current on the coils in group 2, the current of coil 3 adds as much as coil 1. The currents on coils 6 and 7 are subtracted as much as half this amount. The remaining coils (i.e., coils 2 and 4) cancel the *z-*component of the magnetic field generated by the other coils. The same method can be applied in the *y-*direction because the coils are aligned in a similar manner as in the *x-*direction.

To form gradient in *x-*direction, the zero-gradient current combination is obtained using a pseudo-inverse matrix as follows:

7 $${\varvec{I}}=\left[\begin{array}{c}{i}_{1}\\ {i}_{2}\\ {i}_{3}\\ {i}_{4}\\ {i}_{5}\\ {i}_{6}\\ {i}_{7}\\ {i}_{8}\end{array}\right].$$

To form a gradient in the *x-*direction, the coils in group 1 increase the current intensity, whereas those in group 2 decrease. Defining the current change to ${\alpha i}_{1}$, the current used to form the gradient changes to

8 $$\left[\begin{array}{c}{i}_{1}^{*}\\ {i}_{2}^{*}\\ {i}_{3}^{*}\\ {i}_{4}^{*}\\ {i}_{5}^{*}\\ {i}_{6}^{*}\\ {i}_{7}^{*}\\ {i}_{8}^{*}\end{array}\right]=\left[\begin{array}{c}{i}_{1}+({i}_{1}\alpha )\\ -({\overrightarrow{B}}_{z}/{\tilde{B }}_{z, 2})/2\\ {i}_{3}+({i}_{1}\alpha )\\ -({\overrightarrow{B}}_{z}/{\tilde{B }}_{z, 2})/2\\ {i}_{5}-({i}_{1}\alpha )/2\\ {i}_{6}-({i}_{1}\alpha )/2\\ {i}_{7}-({i}_{1}\alpha )/2\\ {i}_{8}-({i}_{1}\alpha )/2\end{array}\right],$$

where ${i}^{*}$ represents changed current to form the gradient, and ${\overrightarrow{B}}_{z}$ represents the *z-*component of the final magnetic field generated by other coils except coil 2 and 4. In addition, ${\tilde{B }}_{z, 2}$ represents the *z-*component of the magnetic field generated by coil 2 with the unit current.

In the case of the *z-*direction, the coils are grouped using the same method, but the current changes differently to form the gradient because the coils are aligned unlike in the *x-* and *y-*directions. Coils 1–4, and coils 5–8, are grouped, namely, groups 1 and 2, respectively. The current in group 1 increases its intensity as much as ${\alpha i}_{1}$, and the current in group 2 decreases proportionally to ${\alpha i}_{1}$. As the coils in groups 1 and 2 generate different *z-*components of the magnetic field, the coils in group 2 change proportionally to the ratio of this difference. Eventually, the current in group 2 changes the current as much as ${\alpha i}_{1}\times {\tilde{B }}_{z, 1}/{\tilde{B }}_{z, 5}$, where ${\tilde{B }}_{z, 1}$ and ${\tilde{B }}_{z, 5}$ represent the *z-*component of the magnetic field generated by coils 1 and 5 with unit current, respectively. For example, to form the gradient in the *z-*direction, the zero-gradient current combination is calculated as

9 $${\varvec{I}}=\left[\begin{array}{c}{i}_{1}\\ {i}_{2}\\ {i}_{3}\\ {i}_{4}\\ {i}_{5}\\ {i}_{6}\\ {i}_{7}\\ {i}_{8}\end{array}\right].$$

In addition, as we defined the current change as ${\alpha i}_{1}$, the current to form the gradient changes to

10 $$\left[\begin{array}{c}{i}_{1}^{*}\\ {i}_{2}^{*}\\ {i}_{3}^{*}\\ {i}_{4}^{*}\\ {i}_{5}^{*}\\ {i}_{6}^{*}\\ {i}_{7}^{*}\\ {i}_{8}^{*}\end{array}\right]=\left[\begin{array}{c}{i}_{1}+({i}_{1}\alpha )\\ {i}_{2}+({i}_{1}\alpha )\\ {i}_{3}+({i}_{1}\alpha )\\ {i}_{4}+({i}_{1}\alpha )\\ {i}_{5}+({i}_{1}\alpha ){\tilde{B }}_{z, 1}/{\tilde{B }}_{z, 5}\\ {i}_{6}+({i}_{1}\alpha ){\tilde{B }}_{z, 1}/{\tilde{B }}_{z, 5}\\ {i}_{7}+({i}_{1}\alpha ){\tilde{B }}_{z, 1}/{\tilde{B }}_{z, 5}\\ {i}_{8}+({i}_{1}\alpha ){\tilde{B }}_{z, 1}/{\tilde{B }}_{z, 5}\end{array}\right].$$

The gradient can be easily formed using the parameter α, which we proposed as described above. In addition, a systemized strategy with closed-loop in simulation to manipulate the nanorobot clusters is adapted as follows:

1. Investigate magnetic characteristics of a system and nanorobot clusters: magnetic information differs according to the number of coils, core material, coil turn, system dimension, and magnetization, and size of nanorobot clusters.
2. Determine the pathway that nanorobot clusters travel within the region of interest (ROI).
3. Calculate the current set that has zero-gradient (i.e., α = 0) with a required magnetic flux density from Eq. (5). Required magnetic flux density can be determined based on the magnetization characteristics of nanorobot clusters.
4. Calculate new current set to generate the gradient of the magnetic field with parameter α by utilizing Eqs. (8) and (10).
5. Apply the current set and alter the current set according to the pathway.

The procedures from steps 3–5 are repeated until clusters reach the targeted area.

## Supplementary Information

Supplementary Information.

Supplementary Video 1.

Supplementary Video 2.
