# Supplementary material for: Free manipulation system for nanorobot cluster based on complicated multi-coil electromagnetic actuator
Source: Sci Rep. 2021 Oct 5;11:19756. doi: 10.1038/s41598-021-98957-y (PMC8492874; doi:10.1038/s41598-021-98957-y)
Supplement: Supplementary file 1 — Supplementary Information. [file 41598_2021_98957_MOESM1_ESM.pdf]

Supplementary Information

Free manipulation system for nanorobot cluster based on complicated multi-coil electromagnetic actuator

Yun Kim[1], Jun Keun Chae[1], Jong-Hwan Lee[2], Eunpyo Choi[3], Yoon Koo Lee[1], and Jihwan Song[1*]

[1] Department of mechanical engineering, Hanbat National University, Daejeon 34158, Republic of Korea
[2] Center for Convergent Research of Emerging Virus Infection, Korea Research Institute of Chemical Technology, Daejeon, 34114, Republic of Korea
[3] School of Mechanical Engineering, Chonnam National University, Gwangju 61186, Republic of Korea

*Corresponding Author: Electronic mail: jsong@hanbat.ac.kr

Figure Legends

Supplementary Fig. S1. Simulation with a single coil to estimate the range of parameter α and the intensity of the magnetic field. (a) The magnetic flux density at the data point with an increase in current on single cobalt and nickel steel core coils. (b) The domain of a single coil. The dimensions of the single coil are the same as the coils in the electromagnetic actuator system. The magnetic flux density is obtained at the data point the same distance from the coil to the center of the channel of electromagnetic actuator system (i.e., 25.5 mm) (COMSOL Multiphysics 5.4).

Supplementary Fig. S2. Controllable magnetic field in the channel in *y*- and *z*-directions. (a) – (d) The magnetic flux density profile in the channel along the *y*-axis with various values of α. The magnetic flux density at the center of the channel is set to (a) 5, (b) 10, (c) 15, and (d) 20 mT, respectively. (e) – (h) The magnetic flux density profile in the channel along the *z*-axis with various values of α. The magnetic flux density at the center of the channel is set to (a) 5, (b) 10, (c) 15, and (d) 20 mT, respectively.

Supplementary Fig. S3. Controllable magnetic field in the channel using the nickel steel core. (a) – (d) The magnetic flux density profile in the channel along the *x*-axis with various values of α. The magnetic flux density at the center of the channel is set to (a) 2.5, (b) 5, (c) 7.5, and (d) 10 mT, respectively. (e) The

magnetic field in the channel with magnetic flux density of 10 mT for a parameter change of (i) – (iv) 0.25 to 1.00, respectively (COMSOL Multiphysics 5.4).

Supplementary Fig. S4. Spatial control of the nanorobot cluster with various magnetic fields according to the change in parameter α. (a) Three-dimensional control of nanorobot cluster. (b) The average velocity of the nanorobot cluster while traveling (COMSOL Multiphysics 5.4).

Supplementary Fig. S5. Spread locomotion of nanorobot clusters. (a) Domain of the channel labeled with viewpoint. (b) Magnetic flux density for the case of the *x*-directional spread. Targeted direction for spreading is (c) *x*-, (d) *y*-, and (e) *z*-direction (COMSOL Multiphysics 5.4).

Current limit and linearity

Simulations with a single coil using COMSOL Multiphysics 5.4 were conducted to estimate the range of parameter α and the intensity of the magnetic field. After the core saturation, the induced magnetic field is no longer linear to the current, which makes it difficult to predict the magnetic field. Two materials for the core, cobalt steel and nickel steel, are considered, and are also considered in this single-coil simulation. Because these two materials have different magnetic properties, their saturation currents differ. As shown in Supplementary Fig. S1, the nickel steel core saturates at a lower current than the cobalt steel core. Since this difference in the magnetic property, the electromagnetic actuation system with a nickel core uses a lower intensity of the magnetic field because the higher intensity of the magnetic field requires a higher current. The magnetic flux density is linear until approximately 6 A and 10 A in the case of nickel and cobalt steels, respectively. The intensity of the magnetic field and the range of parameter α were chosen based on these results.

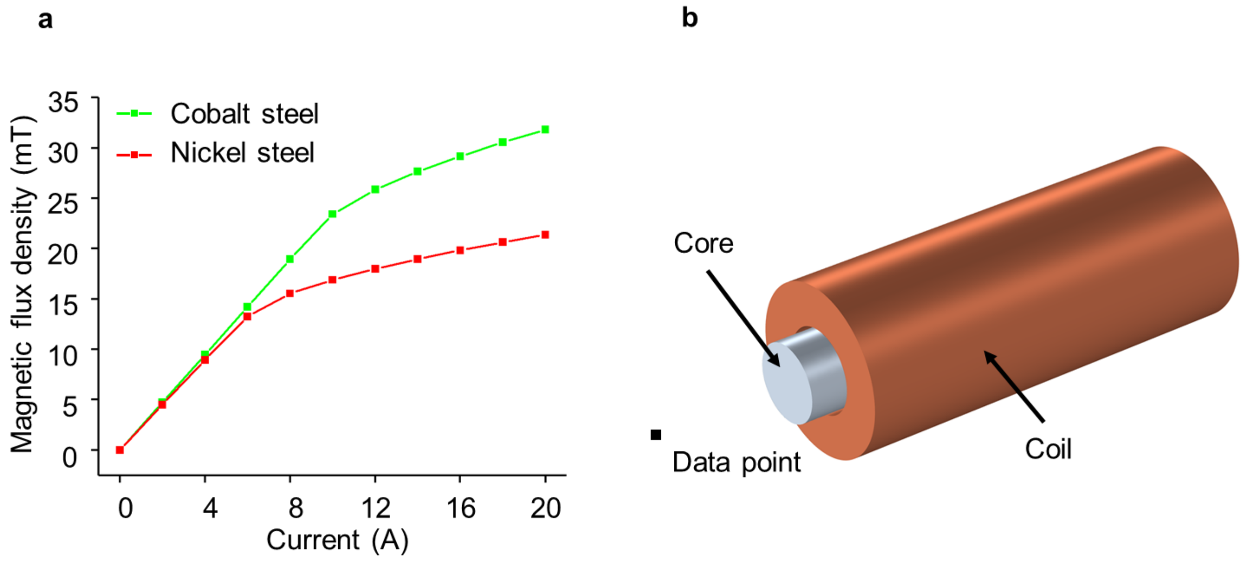

Supplementary Fig. S1. Simulation with a single coil to estimate the range of parameter α and the intensity of the magnetic field. (a) The magnetic flux density at the data point with an increase in current on single

cobalt and nickel steel core coils. (b) The domain of a single coil. The dimensions of the single coil are the same as the coils in the electromagnetic actuator system. The magnetic flux density is obtained at the data point the same distance from the coil to the center of the channel of electromagnetic actuator system (i.e., 25.5 mm) (COMSOL Multiphysics 5.4).

Controllable magnetic field in *y*- and *z*-directions

In the case of the *y*-direction, the magnetic field is formed similar to the *x*-direction, as shown in Supplementary Fig. S2. Four cases, similar to the case of the *x*-direction (i.e., 5, 10, 15, and 20 mT of the magnetic flux densities at the center of the channel) were considered (Supplementary Fig. S2a to d) along the *y*-axis. For 5 mT, the magnetic flux density at the ends of the channel in the *y*-axis varies 4.9 to 3.6 mT at the negative end (i.e., at the position of $y = -5.0$ mm) and it varies to 6.2 mT at the positive end (i.e., at the position of $y = 5.0$ mm) when the parameter α changes 0 to 1.00 (Supplementary Fig. S2a). For 10 mT, the magnetic flux density at the ends of the channel in the *y*-axis varies 9.8 to 7.28 mT at the negative end and is 12.5 mT at the positive end of the channel when the parameter α changes from 0 to 1.00 (Supplementary Fig. S2b). In the case of 15 mT in intensity, the magnetic flux density varies from 14.7 to 10.9 mT at the negative end and to 18.8 mT at the positive end of the channel where α is 0 to 1.00 (Supplementary Fig. S2c). For 20 mT, the magnetic flux density varies from 19.7 to 14.3 mT at the negative end of the channel and to 24.4 mT at the positive end of the channel when α is 0 to 1.00. (Supplementary Fig. S2d). For 5, 10, 15, and 10 mT, the gradient increases from 0 to 0.26, 0 to 0.52, 0 to 0.78, and 0 to 1.00 T/m, respectively, with the increase of α from 0 to 1.00.

In the case of the *z*-direction, the intensity of the magnetic field is the same as in the *x*- and *y*-directions. However, parameter α varies differently (i.e., from 0.0 to 4.0). For 5 mT, the magnetic flux density at the ends of the channel varies from 4.9 to 4.0 mT and to 6 mT at the negative and positive ends of the channel (i.e., at positions of $z = -5$ and 5 mm), respectively, where the parameter α changes from 0.0 to 4.0 (Supplementary Fig. S2e). For 10 mT, it changes from 9.8 to 8.1 mT and 12.0 mT at the ends of the channel

when parameter α changes from 0.0 to 4.0 (Supplementary Fig. S2f). For 15 mT, it changes from 14.9 to 12.1 mT and 18.1 mT at the ends of the channel when α changes from 0.0 to 4.0 (Supplementary Fig. S2g). In the case of 20 mT, the magnetic flux density at the ends of the channel in the *z*-axis varies from 19.7 mT to 16.1 mT at the negative end of the channel and to 24.0 mT at the positive end (Supplementary Fig. S2h). Additionally, gradient increases from 0 to 0.20, 0 to 0.40, 0.60, and 0 to 0.80 T/m when α is 0.0 to 4.0 and the magnetic flux density is 5, 10, 15, and 20 mT, respectively. Regardless of the direction and intensity of the magnetic field, parameter α can simply control the gradient.

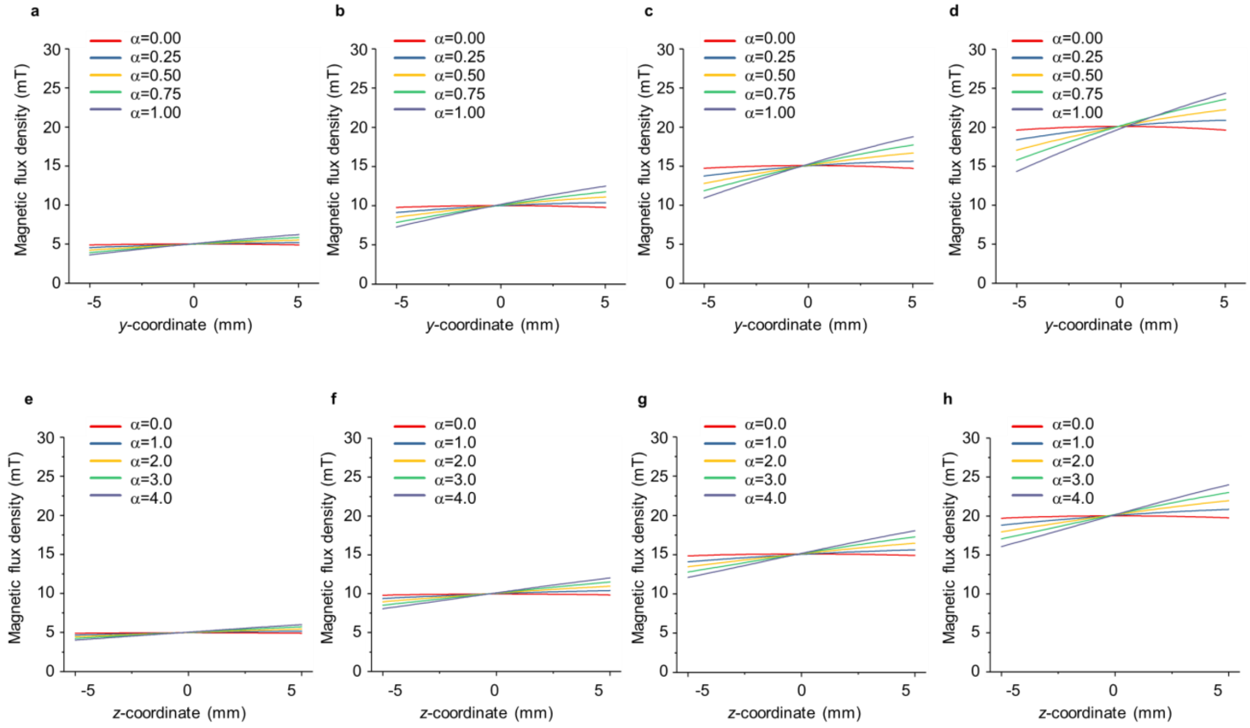

Supplementary Fig. S2. Controllable magnetic field in the channel in *y*- and *z*-directions. (a) – (d) The magnetic flux density profile in the channel along the *y*-axis with various values of α. The magnetic flux density at the center of the channel is set to (a) 5, (b) 10, (c) 15, and (d) 20 mT, respectively. (e) – (h) The magnetic flux density profile in the channel along the *z*-axis with various values of α. The magnetic flux density at the center of the channel is set to (a) 5, (b) 10, (c) 15, and (d) 20 mT, respectively.

Magnetic field with nickel core

To show the adaptability of the formation of the magnetic field and its gradient with parameter α, irrespective of the core materials, not only the cobalt steel for core but a nickel steel are considered for the core. In the case of the nickel steel core, the magnetic flux density at the center of the channel is different from that of the cobalt steel core because it has different magnetic properties from cobalt steel. To generate the magnetic flux density and its gradient with the nickel steel core, α varies from 0 to 1.00 (Supplementary Fig. S3). Although cobalt and nickel steels have different magnetic properties, the gradient of the magnetic field can be simply formed using the proposed parameter. Four cases of magnetic field intensity are considered (i.e., 2.5, 5, 7.5, and 10 mT). In the case of 2.5 mT, the gradient of magnetic field is obtained as 0, 0.04, 0.07, 0.10, and 0.13 T/m when α varies from 0, 0.25, 0.50, 0.75, and 1.00. For 5, 7.5, and 10 mT, the gradient increases from 0 to 0.26 T/m, 0 to 0.39 T/m, and 0 to 0.52 T/m, respectively, with the increase of α from 0 to 1.00.

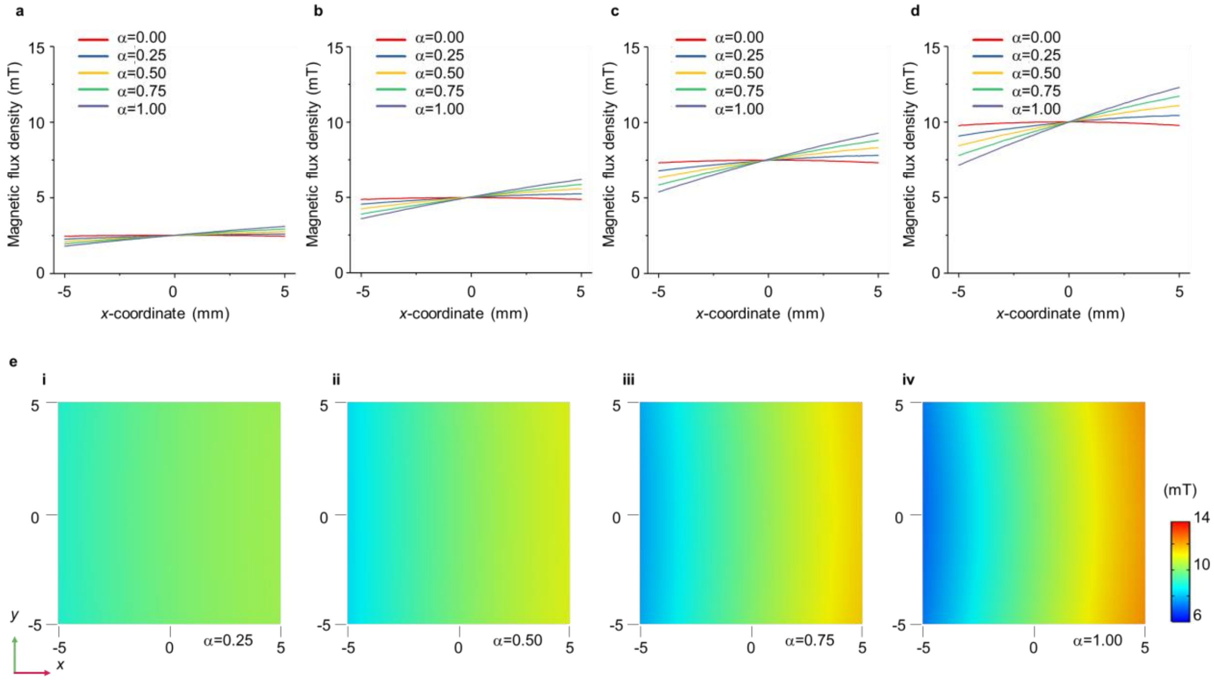

Supplementary Fig. S3. Controllable magnetic field in the channel using the nickel steel core. (a) – (d) The magnetic flux density profile in the channel along the *x*-axis with various values of α. The magnetic flux density at the center of the channel is set to (a) 2.5, (b) 5, (c) 7.5, and (d) 10 mT, respectively. (e) The magnetic field in the channel with magnetic flux density of 10 mT for a parameter change of (i) – (iv) 0.25 to 1.00, respectively (COMSOL Multiphysics 5.4).

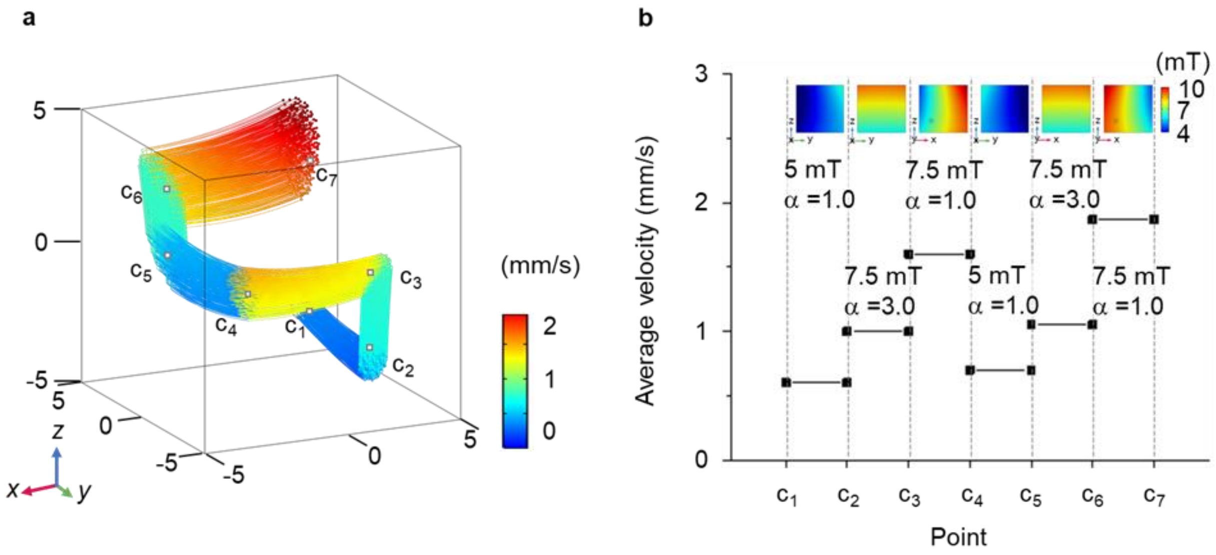

Supplementary Fig. S4. Spatial control of the nanorobot cluster with various magnetic fields according to the change in parameter α. (a) Three-dimensional control of nanorobot cluster. (b) The average velocity of the nanorobot cluster while traveling (COMSOL Multiphysics 5.4).

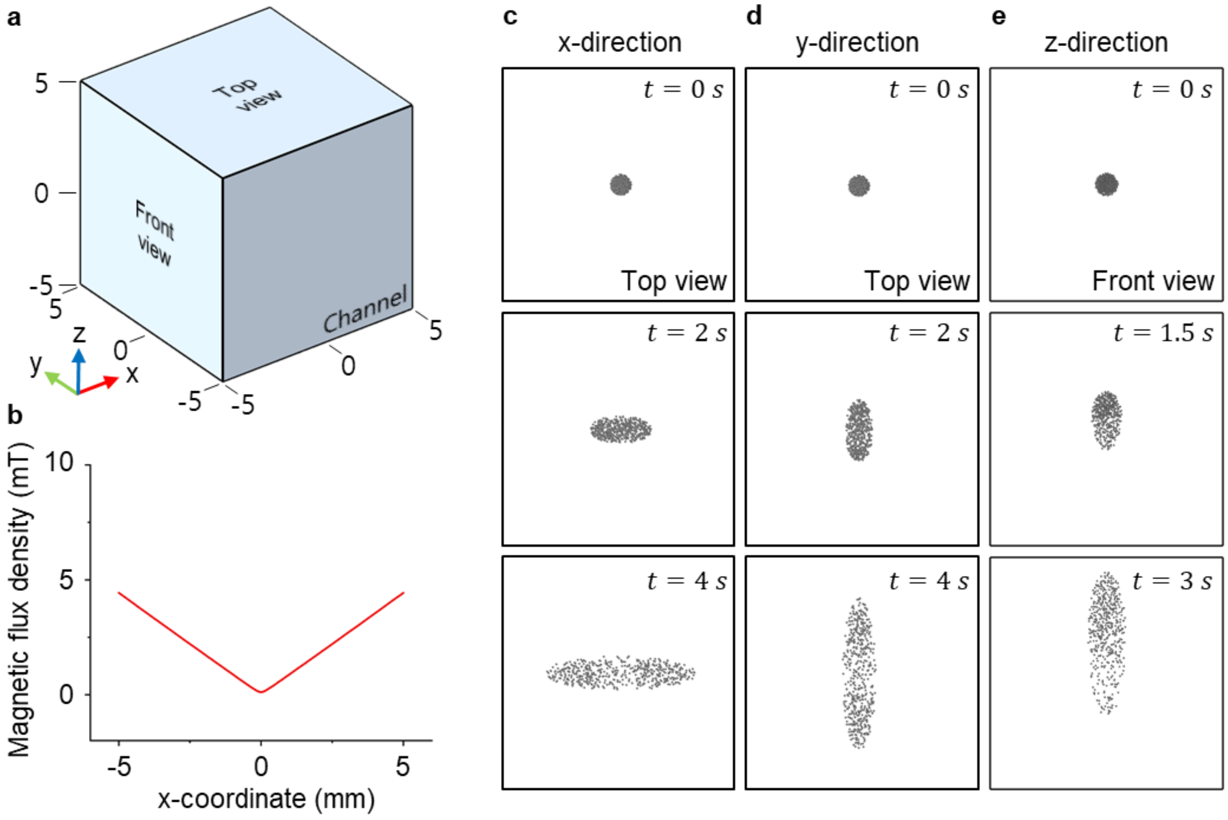

Supplementary Fig. S5. Spread locomotion of nanorobot clusters. (a) Domain of the channel labeled with viewpoint. (b) Magnetic flux density for the case of the *x*-directional spread. Targeted direction for spreading is (c) *x*-, (d) *y*-, and (e) *z*-direction (COMSOL Multiphysics 5.4).
